# Emergence delirium in small animals: a first step towards an objective assessment

**DOI:** 10.3389/fvets.2025.1623761

**Published:** 2025-06-18

**Authors:** Larissa Irina Mattei, Ludovica Chiavaccini, Claudia Spadavecchia, Alessandro Mirra

**Affiliations:** ^1^Department of Clinical Veterinary Medicine, Section of Anaesthesiology and Pain Therapy, Vetsuisse Faculty, University of Bern, Bern, Switzerland; ^2^Department of Comparative, Diagnostic, and Population Medicine, College of Veterinary Medicine, University of Florida, Gainesville, FL, United States; ^3^Department of Clinical Sciences, Faculty of Veterinary Medicine, University of Montreal, Saint-Hyacinthe, QC, Canada

**Keywords:** emergence, delirium, recovery, score, dogs, cats, dysphoria, anesthesia

## Abstract

**Introduction:**

Emergence Delirium (ED) is a condition characterized by agitation, confusion and disorientation that can occur in human patients during recovery from anesthesia. In veterinary medicine, a similar phenomenon is observed, but the frequency of occurrence and the characteristics of symptoms are not yet well defined. This study aimed to identify ED symptoms in dogs and cats, explore their occurrence, and recognize related factors.

**Materials and methods:**

In this prospective observational study anesthesia providers systematically, recorded symptoms observed in patients during the recovery phase following anesthesia. The study was divided into three phases. Phase 1: a group of six veterinary anesthesiologists created a list of symptoms possibly related to ED in small animals.Phase 2: demographic and anesthesia-related data were collected for dogs and cats undergoing general anesthesia at the veterinary teaching hospital of the University of Bern between May 2022 and January 2023. The occurrence of ED symptoms was analyzed and based on the occurrence of the listed ED symptoms, animals were assigned to 2 groups: NED group (No-ED group; less than four symptoms observed); ED group (four or more symptoms observed). Phase 3: a logistic regression analysis was performed to explore potential associations between ED occurrence and subject or anesthesia related factors.

**Results:**

Phase 1: based on expert agreement, six symptoms were selected: nystagmus, paddling, opisthotonos, uncoordinated/violent movement, vocalization, and biting. Phase 2: data were recorded from 184 animals (139 dogs, 45 cats). The most common ED symptoms occurring were uncoordinated movement (41%), vocalization (36%) and paddling (30%). More than half of the study population (104/184, 57%) showed at least one symptom of ED; 14% (26/184) of the animals were included in the ED group. Phase 3: no association between subject or anesthesia related factors and the occurrence of ED was found in the univariable analysis.

**Conclusion:**

Based on the proposed list of symptoms, post anesthetic ED seems to occur frequently in small animals. The observations conducted in the present study can help further evaluation of this phenomenon in dogs and cats.

## 1 Introduction

Emergence delirium (ED) is a condition that can occur as patients wake up from general anesthesia, associated with increased morbidity, mortality and resource utilization in human medicine (1). It is typically characterized by symptoms such as hallucinations and confusion and can be manifested with restlessness, moaning, involuntary physical activity and thrashing (2).

The phenomenon was first described in humans in 1961 (3). An occurrence of 5.3% was reported, which is similar to rates presented in recent publications, ranging from 5 to 10% (4). Since then, numerous reports, particularly on pediatric patients, have been published, but globally recognized guidelines for its recognition, prevention, and treatment are still lacking (1, 2, 5, 6). Several factors have been found to be associated with the occurrence of ED, among which age, mental comorbidity, drugs (especially benzodiazepines and opioid), long surgery duration and inadequate pain therapy (1, 2). The pathophysiology of delirium in the context of anesthesia is multifactorial, involving alterations in neurotransmitter balance (particularly acetylcholine and dopamine), neuroinflammation, disrupted sleep–wake cycles, and impaired cortical connectivity, all of which may be triggered or exacerbated by anesthetic agents and perioperative stress (7–9). However, many aspects regarding its underlying causes remain unclear, posing challenges to the development of effective management strategies.

In veterinary medicine, even less is known about ED prevalence, symptoms and predisposing factors. Although previous reports suggest that benzodiazepine (10) and opioid (11, 12) may negatively impact recovery quality, no clear association between ED and anesthetic/analgesic drugs has been established yet. The 2020 AAHA (American Animal Hospital Association) “Anesthesia and Monitoring Guidelines for Dogs and Cats” describes ED as an uncontrolled, uncoordinated thrashing of the patient during the regain of consciousness, leaving its identification open to subjective judgment (13). Predisposing factors are also unknown.

Given the lack of standardized criteria for ED recognition and the paucity of reports about potential predisposing factors in veterinary medicine, the present study aimed to: 1) define objective symptoms of ED in dogs and cats, 2) investigate their appearance in animals anesthetized for clinical procedures, 3) identify factors possibly associated with their occurrence.

## 2 Materials and methods

A prospective observational study was conducted, divided into three phases.

### 2.1 Phase 1: definition of “emergence delirium” (ED)

An initial list of symptoms potentially indicating ED in dogs and cats was drafted based on personal experience and previous literature (6, 12, 14, 15). Following consensus of six veterinary anesthesiologists (two residents and four diplomates of the European College of Veterinary Anesthesia and Analgesia, ECVAA), six symptoms were selected to be used in the clinical evaluation of ED occurrence: nystagmus, paddling, opisthotonos, uncoordinated movement, vocalization and biting. Additionally, it was established that a minimum of four symptoms had to be present to classify the phenomenon occurring during the recovery phase as ED.

### 2.2 Phase 2: data collection

Data were collected from clinical cases admitted at the small animal clinic of the University of Bern, between May 2022 and January 2023. An ad-hoc online form made available via a QR code was used (see Supplementary material). Staff members performing general anesthesia were asked to fill it out for each anesthetic event. For the present study, no specific ethical permission was deemed necessary by the responsible authority for animal experiments of the Canton of Bern, Switzerland.

Information was gathered for the following categories:

- Demographic details (e.g., sex, age, breed);
- Preoperative condition (e.g., temperament, American Society of Anesthesia (ASA) physical status);
- Anesthesia and surgery (e.g., type, duration, emergency vs. elective);
- Recovery phase (e.g., time to extubation);
- Occurrence of ED symptoms according to the predefined list (from moment of extubation to 30 min thereafter);
- Administration of opioid receptor agonists and/or benzodiazepines as part of the anesthetic protocol.

Data collected through the online forms were directly exported into a Microsoft Excel file.

### 2.3 Statistical analysis

For the descriptive analysis, dogs and cats were categorized into two groups, depending on the number of symptoms observed: animals showing 0–3 symptoms were assigned to the NED group (No-ED group) and animals showing 4–6 symptoms were assigned to the ED group.

### 2.4 Inferential analysis

The present study employed the penalized maximum likelihood logistic regression method proposed by Firth for rare events (16), utilizing a permissive *p*-value threshold of > 0.20 for univariable screening of various explanatory variables. Continuous variables were assessed for linear relationships with the log odds of the outcome using the Lowess smoothing function. In cases when linearity assumptions were not met, variables were stratified based on biological rationale and population distribution for further analysis. Predictors with at least 10 occurrences were retained for analysis. Backward elimination was performed, and the likelihood-ratio test was used to assess the contribution of any subset of parameters. The final multivariable model was constructed, with all variables having a statistical significance at *p* ≤ 0.05. If a variable affected another variable’s coefficient by more than 20%, it was considered a confounder and was forced into the model. All interactions were tested for significance. Significant variables were expressed as odds ratios with corresponding 95% Wald intervals and *p* values. The Hosmer–Lemeshow statistic was employed to evaluate the overall fit of the model. Given the rarity of the outcome and the limited sample, the feline and canine populations were then analyzed together, including only predictors meeting inclusion criteria in either species during univariate analysis. The need for restraint at recovery, sedative use prior to anesthesia, preventive sedation at recovery and ASA physical status classification was retested with univariable analysis. The same criteria previously discussed were used to build and test the multivariable model, ensuring the robustness and generalizability of the findings.

## 3 Results

A total of 184 cases were collected (139 dogs and 45 cats). The main results are reported below.

### 3.1 Demographic data, preoperative information, information regarding anesthesia, surgery, and recovery

Detailed tables containing the demographic data can be found in the Supplementary material.

Among dogs, 136/139 (98%) had an ASA status of 3 or lower. Six were Border Collies: four received sedation during the recovery phase, 3 of which in a preventive way. Twenty dogs of the Retriever breeds (Golden Retriever, Labrador Retriever) were also included: six of them (30%) received sedation during the recovery phase, only one in a preventive way. Most of the dogs needing physical restraint during the recovery phase were allocated in the NED group 18/23 (78%); the majority of them (13/23; 57%) received a sedative drug.

Among cats, 44/45 (98%) had an ASA status of 3 or lower. Only 7/45 (16%) were sedated, 4 of which received it preventively. Seven cats out of 45 (16%) required physical restraint; most were allocated in the NED group (5/7; 71%).

### 3.2 Symptoms

**Figure 1:**
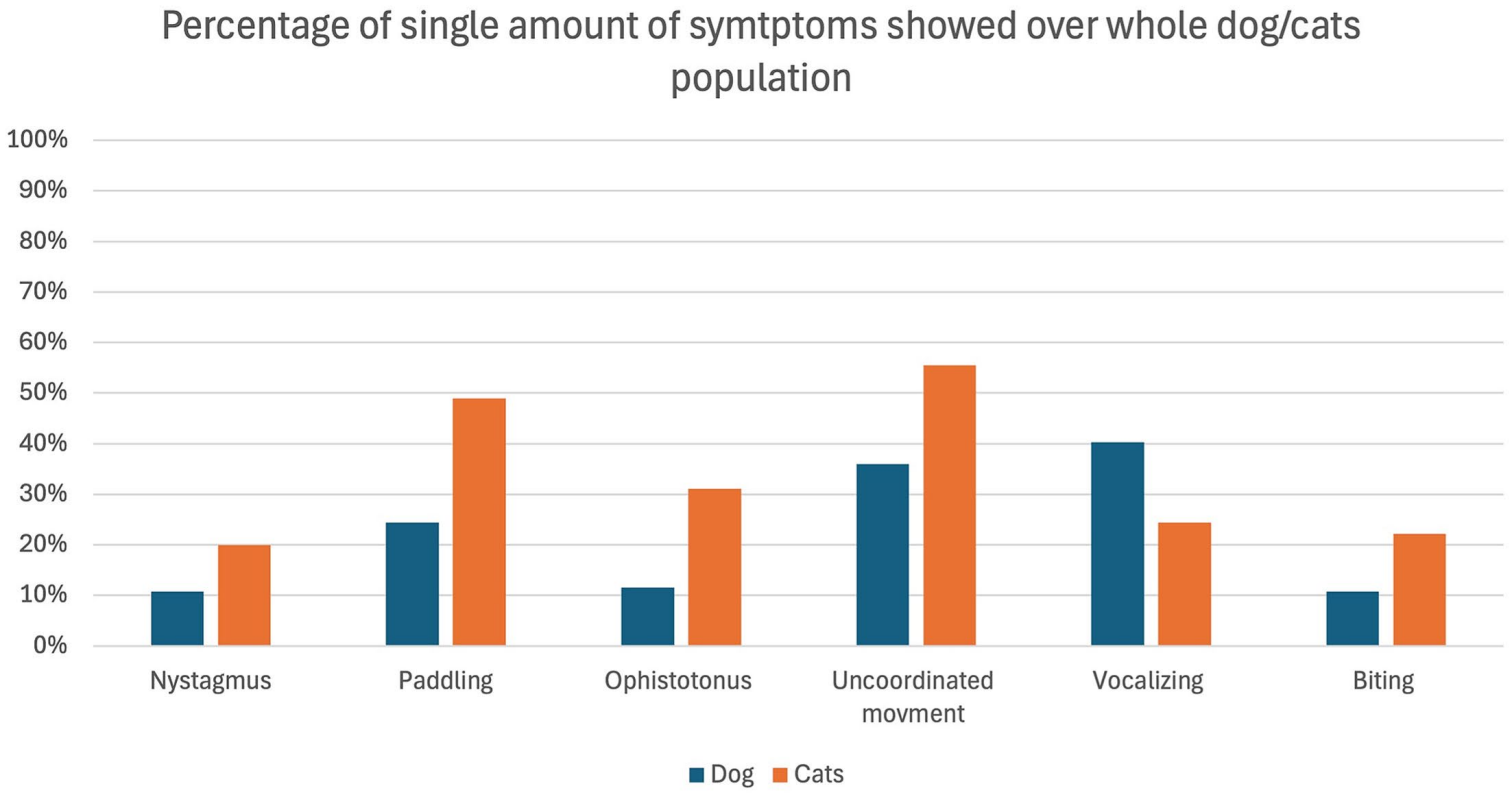
Percentage of dogs (blue) and cats (orange) showing the individual symptoms in relation to the whole population of dogs (*n* = 139) and cats (*n* = 45) included in the study.

**Figure 2:**
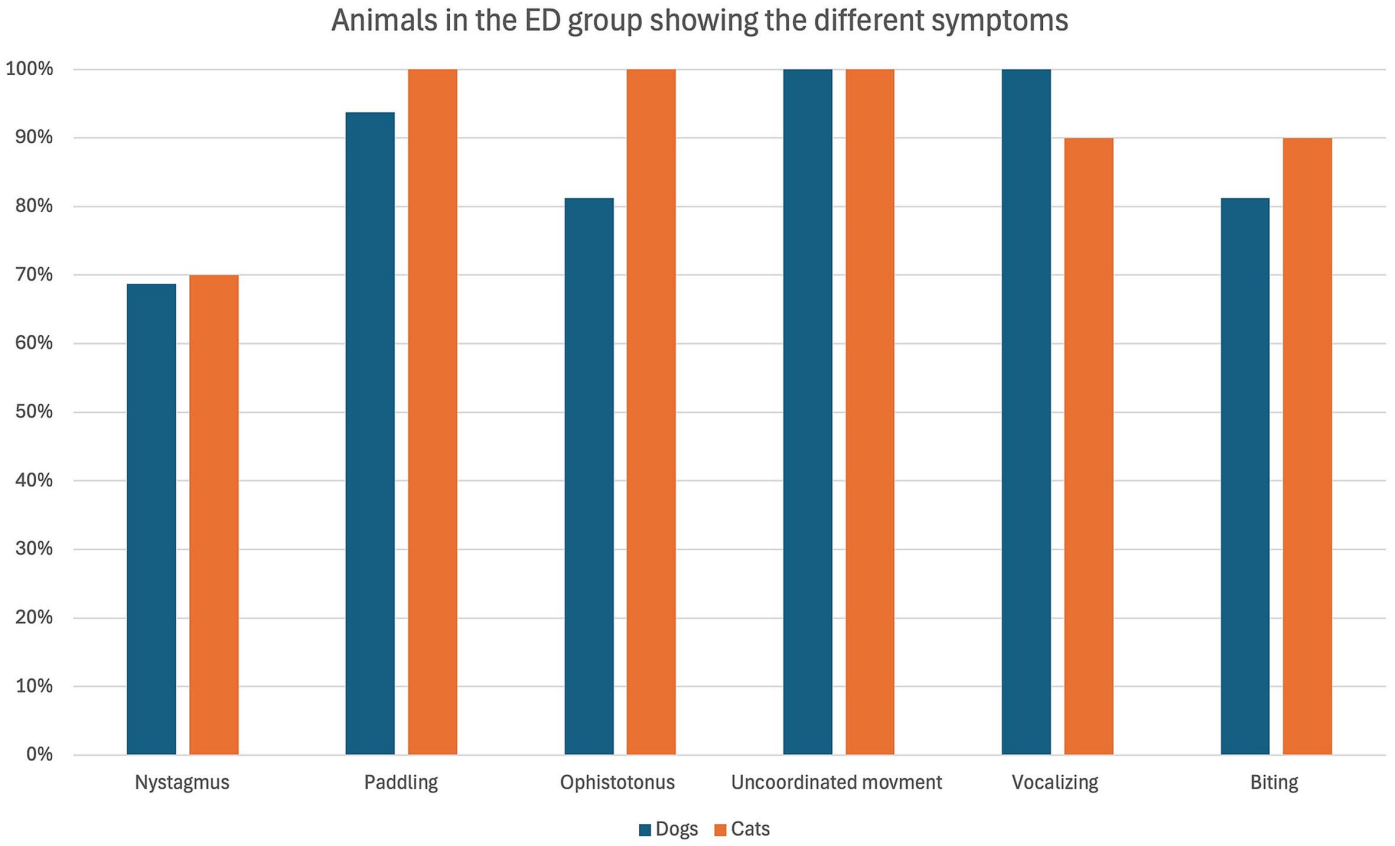
Percentage of dogs (blue) and cats (orange) belonging to the ED group (16 dogs and 10 cats in total) showing the defined symptoms.

**Figure 3:**
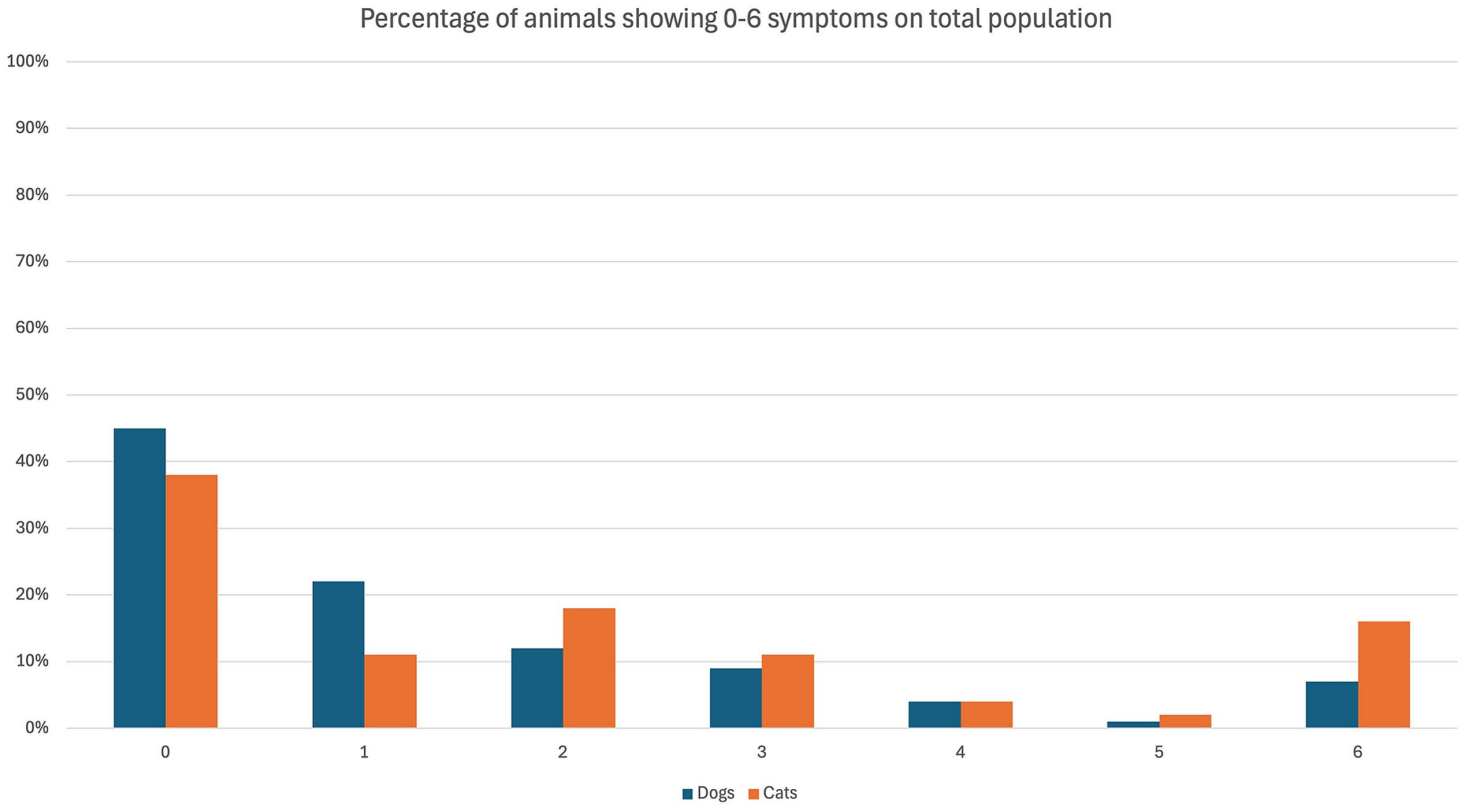
Symptoms of emergence delirium (ED) observed in dogs (blue) and cats (orange) in the course of the present study. The percentage refers to the overall population of the specific species.

The overall occurrence of ED symptoms is reported in Figure 1, while in Figure 2 symptoms occurring in the ED group only are reported. Figure 3 illustrates the percentage of animals showing 0–6 symptoms over the whole study population; 10/139 (7%) of the dogs and 7/45 (16%) of the cats (17/184 (9%) of the total population) showed all six symptoms.

### 3.3 Inferential analysis

Following univariable analysis, whether the dog received any sedation before presentation for anesthesia (Wald = 1.83, *p* = 0.18) and the need to restrain the animal (Wald = 3.03, *p* = 0.08) were the only variables meeting the criteria for inclusion in the multivariable model. However, the final model did not reach significance (Wald = 3.97, *p* = 0.14).

When considering the cat dataset, whether the cat’s ASA physical status was ≥ III (Wald = 1.65, *p* = 0.20) and the preventive use of postoperative sedation (Wald = 2.92, *p* = 0.09) met the criteria for inclusion in the multivariable model. However, they lost significance in the model (Wald = 3.82, *p* = 0.15). When considering both populations together, we found no association in the univariable analysis.

## 4 Discussion

In the present study, more than half of the study cohort showed at least one symptom, while 14% (26/184) were considered to display ED (Group ED: 22% (10/45) of the cats; 12% (16/139) of the dogs).

Viswanath et al. reported an ED prevalence of 5–10% in human medicine (1). The similarity in the percentage of animals showing all six symptoms (9%) suggests that a cluster of symptoms should probably be used to diagnose ED specifically. In present study, it was arbitrarily decided to consider a minimum of 4 coexisting symptoms as necessary to define ED. This approach resembles that of several pain assessment tools like the Glasgow Composite Measure Pain Scale (CMPS) (17), necessitating a minimum number of symptoms or behaviors to be present to diagnose pain in animals. Symptoms of ED considered in the present study had been previously described for humans (2, 3, 18, 19). Restlessness, vocalization, agitation, thrashing and non-purposeful movements were reported by Cole et al. in pediatric patients (2). In contrast, vocalization, excitement, and paddling were included in the Simple Descriptive Scale (SDS) described by Lozano et al. (20) and previously applied to dogs (21).

The most frequent symptom identified in the present study was “uncoordinated movement,” which was present in all the animals of the ED group. It was followed by “paddling” and “vocalizing” which were both present in 96% (25/26 animals) of the ED group.

This is an interesting aspect, as this clinical manifestation is very similar to the one described in children of preschool age (22, 23). Interestingly, all symptoms except “vocalizing” occurred more frequently in cats than dogs. This makes the identification of ED in dogs more obvious compared to cats, which shows it in a more silent way. The lack of reports on the incidence of ED or quality of recovery in cats, and the fact that they manifest ED in a more “silent” way, may indicate a potential underestimation of this phenomenon, which appears to have been overlooked until now.

### 4.1 Demographic data

Within the study population, 55% (76/139) of the dogs and 62% (28/45) of the cats showed at least one symptom, but only 12% (16/139) dogs, and 22% (10/45) cats were categorized in group ED. Our results differ from those of Jones et al. (21), who reported a low recovery score in about one-third (29.1%) of the dogs. However, a direct comparison cannot be made due to the different approaches evaluating recovery quality. In particular, Jones et al. (21) used a simple descriptive scale (SDS) [as previously described by Lozano et al. (20)] and the level of Consciousness-Richmond Agitation and Sedation Scale (LOC-RASS) [as previously described by Sessler et al. (24)]. In the present study, a purpose-made scale was applied.

It is commonly suggested that certain dog breeds may experience rougher recovery phases. However, limited evidence supports this claim. According to a study by Kongara et al. (12), Alaskan Malamutes, Siberian Huskies and Labrador Retrievers are at higher risk of experiencing dysphoria due to the prevalence of a specific allele variant (C-15 A). However, Jones et al. (21) did not confirm this hypothesis, nor did the results presented here (only 20% of the Retriever dogs were in the ED group). Nonetheless, the limited number of animals per breed in this study poses challenges in interpreting the results. Further investigations with more animals should be conducted to identify possible breed predispositions.

In human medicine, there is no agreement on whether sex influences the prevalence of ED. While some studies report an increased prevalence in male individuals (14, 19, 22, 25, 26), others do not support it (27). In veterinary medicine, Jones et al. (21) could not find any evidence suggesting that sex influences recovery quality. Similar results were found in the present study.

Age has been recognized as a factor contributing to the development of ED in humans, with a higher likelihood present in pediatric patients (5, 22, 28, 29). The specific reasons for the increased incidence in children remain unclear. Some authors proposed that the combination of preschool age and the use of inhalants might act as a risk factor for ED. (1, 30, 31) Moreover, a higher occurrence of ED in preschool children compared to their older counterparts has also been suggested to be linked to the higher distress young individuals have in waking up in an unfamiliar environment (28, 30), which could resemble the situation occurring in small animals. In dogs, Jones et al. (21) could not find any evidence supporting the potential contribution of age to the development of poor recovery quality. In our study, animals in the ED group (both dogs and cats) were younger, but no statistical or clinically meaningful difference was found.

### 4.2 Preoperative information

Different studies in human medicine investigated the possible correlation between ASA status and the appearance of ED; however, none could be confirmed (30–32).

Jones et al. found that an ASA status of III or higher was associated with a decrease in the incidence of poor quality of recovery (21). The authors suggest that this could be due to the slower metabolization of the anesthetics in these patients, allowing a slower and calmer recovery phase (21). In the present study, only four animals (3 dogs and 1 cat) with an ASA status above III were included, precluding further evaluation of this condition.

A factor shown to favor ED occurrence in children is anxiety (e.g., sleep disturbance, eating disturbances, aggression against authority) during the preoperative phase (33). In the present study, no clear link between preoperative behavior and the occurrence of ED symptoms was found.

Pain could also affect the recovery phase. There is no evidence in the literature, but distinguishing between painful conditions and ED during the recovery phase can be difficult. Methods that could help to differentiate pain from emergence delirium may include the use of pain assessment tools—such as the Visual Analogue Scale (VAS) employed in our study—or exclusion-based therapy through the administration of analgesic medications. However, distinguishing between the two phenomena remains challenging. In veterinary medicine, Jones et al. could not show any statistical significance between poor quality of recovery and painful procedures (21). Similarly, in our study, preoperative pain (as assessed by the VAS) did not seem to be linked with the development of ED (77% of the patients who showed ED had a VAS between 0 and 3).

### 4.3 Anesthesia and surgery

In human medicine, benzodiazepines have been reported to increase the risk of ED (34, 35), and one study in veterinary medicine seems to support this hypothesis (10). The administration of opioids has also been reported to be linked to a worse recovery quality (12). However, the word dysphoria is often used in this case, referring to a phenomenon called “opioid dysphoria,” not further characterized in small animals (12, 13). In the present study, most animals received opioids, while only a few received benzodiazepines; thus, any attempt to statistically evaluate the contribution of these drugs to ED occurrence would be uninformative.

The use of TIVA has been reported to be a protective factor for the development of ED in humans when compared with sevoflurane (19, 34, 36). One explanation for this could lie in the pharmacokinetics of the injectable anesthetics (propofol), which could lead to a slower recovery phase and, thus, to a reduced occurrence of ED. (28) In our study, no association between TIVA and the occurrence of ED could be demonstrated. However, this is not surprising due to the low number of animals undergoing TIVA in this study.

Prolonged anesthesia duration seems to predispose to ED in humans (1, 34, 37). To the author’s knowledge, no such evidence is present for small animals, but a clear association between anesthesia duration and compromised recovery has been described in horses (38, 39). No association between the duration of anesthesia and ED occurrence has been found in our study.

### 4.4 Recovery phase

Preventive sedation, administered before the recovery phase, aims to mitigate the risks associated with rapid recoveries, such as self-injury or injury to medical staff. Primarily, alpha-2 agonists such as dexmedetomidine or acepromazine were used for preventive sedation in the present study. The most common reasons for administering preventive sedation included procedures where the occurrence of emergence delirium during recovery could pose a risk to the animal (e.g., ophthalmologic procedures), or specific breed considerations (at the discretion of the anesthetist in charge). In human medicine, rapid recovery from anesthesia can increase the likelihood of postoperative complications, including ED in children (18, 21). A smoother transition from anesthesia to consciousness can be obtained by administering preventive sedation, which is hypothesized to lead to a better recovery quality (21). While guidelines for small animals are lacking, a recent study in dogs showed better recovery quality after a preventive administration of low-dose dexmedetomidine compared to saline (40). Preventive sedation could help to reduce ED, but further studies should be conducted before drawing conclusions.

In this study, 15/133 (12%) dogs and 3/44 (7%) cats were sedated preventively. Those animals were not included in further statistical analysis as we considered preventive sedation to impact recovery quality. Interestingly, the majority of the sedated animals did not show ED occurrence (14/15).

In veterinary medicine, the investigation of emergence delirium (ED) is still in its early stages. Its study is pivotal not only to improve the immediate post-operative period, but also to investigate on the potential long-term consequences. This is great concern also in human medicine (41), and could represent an interesting avenue for future research.

## 5 Limitations

The present study has several limitations. First, the symptoms were selected based on the personal experience of four ECVAA diplomates and two residents, as well as information present in literature, both from human and veterinary medicine. However, applying a systematic approach such as the Delphi method for selecting the symptoms might have led to a more robust outcome (42). Second, opioid dysphoria can cause symptoms similar to those selected to define ED. The differentiation between them is challenging in humans and becomes even more complex in veterinary patients, where verbal communication (key for the recognition of dysphoria) is not possible. This limitation necessitates reliance on indirect indicators, further complicating accurate assessment. Evaluation of the electroencephalographic activity has been used in human medicine to help reducing the occurrence of ED (9, 43). However, clear guidelines have yet to be established, and its application in animals remains in the early stages. Further research should focus on evaluating electroencephalographic parameters as potential tools to reduce the occurrence of emergence delirium (ED) in animals. Importantly, an objective method for identifying ED should first be developed; this study aims to address that need. Third, the animals were anesthetized by different anesthetists with different experiences, and no standardized protocols were used. However, this was done to mirror a clinical scenario. Fourth, no sample size calculation was performed; nonetheless, the study was intended to provide a first analysis of the phenomenon. Fifth, for the inferential analysis, we defined as animals having ED only those showing at least four symptoms. However, this decision is arbitrary and could have influenced the results.

## 6 Conclusion

Postanesthetic ED seems to be prevalent in small animals and may be under-reported, especially in cats. The results of the present study can help to better characterize it and contribute to the development of species-specific scores. A clinical tool for its diagnosis is needed, and further studies are required to better understand its causes and possible preventive measures.

## Data Availability

The original contributions presented in the study are included in the article/Supplementary material, further inquiries can be directed to the corresponding author.
