# Supplementary material for: Emergence delirium in small animals: a first step towards an objective assessment
Source: Front Vet Sci. 2025 Jun 18;12:1623761. doi: 10.3389/fvets.2025.1623761 (PMC12217935; doi:10.3389/fvets.2025.1623761)
Supplement: Supplementary file 1 [file Table_1.docx]

**Table 1 Dogs. Categories used for descriptive and inferential analysis.**

| **Category** | **Descriptive Analysis** | **Inferential Analysis** |
| --- | --- | --- |
| **Demographic Data** | | |
| Species | Dogs | Dogs |
| Breed | Mixed Breed | Others |
|  | Others |  |
|  | Retrievers (Labrador and Golden) | Retrievers |
|  | Border Collies | Collies |
| Sex | Male | Male |
|  | Male castrated | Male castrated |
|  | Female | Female |
|  | Female castrated | Female castrated |
| Age | MEAN | <1 year = pediatric |
|  | SD | >/=1year- >/=75% life span = adult |
|  |  | > 75% life span =geriatric |
| **Preoperative information** | | |
| Behaviour | Calm | Calm |
|  | Not Calm | Not Calm |
|  | Anxious |  |
| ASA | 1 | 1-2 |
|  | 2 |  |
|  | 3 | 3-5 |
|  | 4 |  |
|  | 5 |  |
| VAS | 0-3 | 0-4 no pain |
|  | 4-7 | 5-10 pain |
|  | >7 |  |
| **Anesthesia and Surgery** | | |
| Urgency of procedure | Elective | Elective |
|  | Emergency | Emergency |
| Type of procedure | Surgery | Surgery |
|  | Diagnostic no brain | Diagnostic |
|  | Diagnostic brain |  |
| Type of surgery | Orthopaedics | Orthopaedics |
|  | Abdominal | Abdominal |
|  | Others | Others |
| Duration anesthesia | <1 | >1h =0 |
|  | 1-2h | 1-2h = 1 |
|  | 2-3h | 2-3h =2 |
|  | longer | 3-4h=3 |
|  | longer | >4 h =4 |
| Critical Drugs | Opioids | Opioids: |
|  |  | Intraoperative opioids |
|  |  | Full mu agonists |
|  | Benzodiazepines | Benzodiazepines |
|  |  | ACP |
|  |  | TIVA vs Inhalant |
|  |  | Locoregional |
| **Recovery Phase** | | |
| Time between end of general anaesthesia and extubation | within 3 min | within 3 min |
|  | 3 -10 min | 3 -10 min |
|  | 11-20 min | 11-20 min |
|  | > 20 min | > 20 min |
| Additional measures | Emergency extubation | Emergency extubation |
|  | Physical restrain | Physical restrain |
|  | Preventive sedation | Preventive sedation |
|  | Sedation | (Not analyzed) |

**Table 2 Cats. Categories used for descriptive and inferential analysis.**

| **Category** | **Descriptive Analysis** | **Inferential Analysis** |
| --- | --- | --- |
| **Demographic Data** | | |
| Species | Cats | Cats |
| Breed | European short hair | European short hair |
|  | Others | Others |
| Sex | Male | Male |
|  | Male castrated | Male castrated |
|  | Female | Female |
|  | Female castrated | Female castrated |
| Age | MEAN | <1 year Pediatric =0 |
|  | SD | >/=1 year – 10 years adult =1 |
|  |  | >/= 10 years geriatric =2 |
| **Preoperative information** | | |
| Behaviour | Calm | Calm |
|  | Not Calm | Not Calm |
|  | Anxious |  |
| ASA | 1 | 1-2 |
|  | 2 |  |
|  | 3 | 3-5 |
|  | 4 |  |
|  | 5 |  |
| VAS | 0-3 | 0-4 no pain |
|  | 4-7 | 5-10 pain |
|  | >7 |  |
| **Anesthesia and Surgery** | | |
| Urgency of procedure | Elective | Elective |
|  | Emergency | Emergency |
| Type of procedure | Surgery | Surgery |
|  | Diagnostic no brain | Diagnostics |
|  | Diagnostic brain |  |
| Type of surgery | Orthopaedics | Orthopaedics |
|  | Abdominal | Abdominal |
|  | Others | Others |
| Duration anesthesia | <1 | <2h |
|  | 1-2h |  |
|  | 2-3h | >2h |
|  | longer |  |
| Critical Drugs | Opioids | Opioids: |
|  |  | Intraoperative opioids |
|  |  | Full mu agonists |
|  | Benzodiazepines | Benzodiazepines |
|  |  | ACP |
|  |  | TIVA vs Inhalant |
|  |  | Locoregional |
| **Recovery Phase** | | |
| Time between end of general anaesthesia and extubation | within 3 min | ≤10 min |
|  | 3 -10 min |  |
|  | 11-20 min | >11 min |
|  | > 20 min |  |
| Additional measures | Emergency extubation | Emergency extubation |
|  | Physical restrain | Physical restrain |
|  | Preventive sedation | Preventive sedation |
|  | Sedation | (Not analyzed) |
