# Supplementary material for: Emergence delirium in small animals: a first step towards an objective assessment
Source: Front Vet Sci. 2025 Jun 18;12:1623761. doi: 10.3389/fvets.2025.1623761 (PMC12217935; doi:10.3389/fvets.2025.1623761)
Supplement: Supplementary file 3 [file Table_3.docx]

**Table 3 Dogs. Demographic and preoperative data, information regarding anesthesia, surgery and recovery phase.** Data reported for dogs (from left to right) divided in the NED group and in the ED group. Round brackets refer to the percentage of animals compared to the row; square brackets refer to the percentage of animals compared to the column (total, except for "Type of surgery", for which the percentages refer only to cases undergoing a surgical procedure). * Categories added after screening the first 43 questionaries: the total number of animals needed to be adapted for these rows.

|  |  | **NED** | **ED** | **All Dogs** |
| --- | --- | --- | --- | --- |
| **Demographic Data** | | | | |
| Species | Dogs | 123 (88%) | 16 (12%) | 139 |
| Breed | Mixed Breed | 17 (100%) | 0 (0%) | 17 [12%] |
|  | Retrivers (Labrador and Golden) | 16 (80%) | 4 (20%) | 20 [14%] |
|  | Border Collies | 6 (100%) | 0 (0%) | 6 [4%] |
|  | Others | 84 (87%) | 12 (13%) | 96 [69%] |
| Sex | Male | 30 (83%) | 6 (17%) | 36 [26%] |
|  | Male castrated | 30 (83%) | 6 (17%) | 36 [26%] |
|  | Female | 28 (93%) | 2 (7%) | 30 [22%] |
|  | Female castrated | 35 (95%) | 2 (5%) | 37 [27%] |
| Age | MEAN | 5.73 | 4.44 | 5.58 |
|  | SD | 3.7 | 3.97 | 3.74 |
| **Preoperative information** | | | | |
| Behaviour | Calm | 59 (91%) | 6 (9%) | 65 [47%] |
|  | Not Calm | 27 (77%) | 8 (23%) | 35 [25%] |
|  | Anxious | 37 (95%) | 2 (5%) | *39/104 [38%] |
| ASA | 1 | 29 (91%) | 3 (9%) | 32 [23%] |
|  | 2 | 78 (89%) | 10 (11%) | 88 [63%] |
|  | 3 | 13 (81%) | 3 (19%) | 16 [12%] |
|  | 4 | 1 (100%) | 0 (0%) | 1 [1%] |
|  | 5 | 2 (100%) | 0 (0%) | 2 [1%] |
| VAS | 0-3 | 84 (87%) | 12 (13%) | 96 [69%] |
|  | 4-7 | 33 (94%) | 2 (6%) | 35 [25%] |
|  | >7 | 6 (75%) | 2 (25%) | 8 [6%] |
| **Anaesthesia and Surgery** | | | | |
| Urgency of procedure | Elective | 106 (88%) | 14 (12%) | 120 [86%] |
|  | Emergency | 17 (89%) | 2 (11%) | 19 [14%] |
| Type of procedure | Surgery | 64 (91%) | 6 (9%) | 70 [50%] |
|  | Diagnostic no brain | 48 (84%) | 9 (16%) | 57 [41%] |
|  | Diagnsotic brain | 11 (92%) | 1 (8%) | 12 [9%] |
| Type of surgery | Orthopaedics | 22 (92%) | 2 (8%) | 24 [34%] |
|  | Abdominal | 11 (85%) | 2 (15%) | 13 [19%] |
|  | Others | 31 (94%) | 2 (6%) | 33 [47%] |
| Duration anaesthesia | <1 | 18 (100%) | 0 (0%) | 18 [13%] |
|  | 1-2h | 50 (85%) | 9 (15%) | 59 [42%] |
|  | 2-3h | 25 (81%) | 6 (19%) | 31 [22%] |
|  | longer | 30 (97%) | 1 (3%) | 31 [22%] |
| Critical Drugs | Opioids | 121 (89%) | 15 (11%) | 136 [98%] |
|  | Bezodiazepines | 13 (87%) | 2 (13%) | 15 [11%] |
|  |  |  |  |  |
|  | TIVA | 5 (83%) | 1 (17%) | 6 [4%] |
| **Recovery Phase** | | | | |
| Extubation Time | within 3 min | 17 (81%) | 4 (19%) | 21 [15%] |
|  | 3 -10 min | 70 (89%) | 9 (11%) | 79 [57%] |
|  | 11-20 min | 26 (90%) | 3 (10%) | 29 [21%] |
|  | > 20 min | 10 (100%) | 0 (0%) | 10 [7%] |
| Aditional measures | Emergency extubation | 5 (83%) | 1 (17%) | 6 [4%] |
|  | Physiscal restrain | 18 (78%) | 5 (22%) | 23 [17%] |
|  | Sedation | 29 (88%) | 4 (12%) | 33 [24%] |
|  | Preventive sedation | 14 (93%) | 1 (7%) | *15/133 [12%] |

**Table 4 Cats. Demographic and preoperative data, information regarding anesthesia, surgery and recovery phase.** Data reported for cats (from left to right) divided in the NED group and in the ED group. Round brackets refer to the percentage of animals compared to the row; square brackets refer to the percentage of animals compared to the column (total, except for "Type of surgery", for which the percentages refer only to cases undergoing a surgical procedure). * Categories added after screening the first 43 questionaries: the total number of animals needed to be adapted for these rows.

|  |  | **NED** | **ED** | **All Cats** | |
| --- | --- | --- | --- | --- | --- |
| **Demographic Data** | | | | | |
| Species | Cats | 35 (78%) | 10 (22%) | | 45 |
| Breed | ESH | 26 (76%) | 8 (24%) | | 34 [76%] |
|  | Others | 9 (82%) | 2 (18%) | | 11 [24%] |
| Sex | Male | 3 (100%) | 0 (0%) | | 3 [7%] |
|  | Male castrated | 15 (83%) | 3 (17%) | | 18 [40%] |
|  | Female | 5 (71%) | 2 (29%) | | 7 [16%] |
|  | Female castrated | 12 (71%) | 5 (29%) | | 17 [38%] |
| Age | MEAN | 6.82 | 4.05 | | 6.19 |
|  | SD | 5.42 | 4.15 | | 5.25 |
| **Preoperative information** | | | | | |
| Behaviour | Calm | 17 (81%) | 4 (19%) | | 21 [47%] |
|  | Not Calm | 9 (69%) | 4 (31%) | | 13 [29%] |
|  | Anxious | 9 (82%) | 2 (18%) | | *11/37 [30%] |
| ASA | 1 | 6 (85%) | 1 (14%) | | 7 [16%] |
|  | 2 | 17 (68%) | 8 (32%) | | 25 [56%] |
|  | 3 | 11 (92%) | 1 (8%) | | 12 [27%] |
|  | 4 | 1 (100%) | 0 (0%) | | 1 [2%] |
|  | 5 | 0 (0%) | 0 (0%) | | 0 [0%] |
| VAS | 0-3 | 21 (72%) | 8 (28%) | | 29 [64%] |
|  | 4-7 | 12 (86%) | 2 (14%) | | 14 [31%] |
|  | >7 | 2 (100%) | 0 (0%) | | 2 [4%] |
| **Anaesthesia and Surgery** | | | | | |
| Urgency of procedure | Elective | 30 (75%) | 10 (25%) | | 40 [89%] |
|  | Emergency | 5 (100%) | 0 (0%) | | 5 [11%] |
| Type of procedure | Surgery | 19 (76%) | 6 (24%) | | 25 [56%] |
|  | Diagnostic no brain | 11 (79%) | 3 (21%) | | 14 [31%] |
|  | Diagnsotic brain | 5 (83%) | 1 (17%) | | 6 [13%] |
| Type of surgery | Orthopaedics | 4 (57%) | 3 (43%) | | 7 [28%] |
|  | Abdominal | 6 (86%) | 1 (14%) | | 7 [28%] |
|  | Others | 9 (82%) | 2 (18%) | | 11 [44%] |
| Duration anaesthesia | <1 | 3 (60%) | 2 (40%) | | 5 [11%] |
|  | 1-2h | 13 (87%) | 2 (13%) | | 15 [33%] |
|  | 2-3h | 6 (86%) | 1 (14%) | | 7 [16%] |
|  | longer | 13 (72%) | 5 (28%) | | 18 [40%] |
| Critical Drugs | Opioids | 33 (79%) | 9 (21%) | | 42 [93%] |
|  | Benzodiazepines | 12 (86%) | 2 (14%) | | 14 [31%] |
|  | TIVA | 1 (100%) | 0(0%) | | 3 [7%] |
| **Recovery Phase** | | | | | |
| Extubation Time | within 3 min | 5 (56%) | 4 (44%) | | 9 [20%] |
|  | 3 -10 min | 20 (87%) | 3 (13%) | | 23 [5%] |
|  | 11-20 min | 8 (73%) | 3 (27%) | | 11 [24%] |
|  | > 20 min | 2 (100%) | 0 (0%) | | 2 [4%] |
| Aditional measures | Emergency extubation | 1 (100%) | 0 (0%) | | 1 [2%] |
|  | Physical restrain | 5 (71%) | 2 (29%) | | 7 [16%] |
|  | Sedation | 5 (71%) | 2 (29%) | | 7 [16%] |
|  | Preventive sedation | 1 (33%) | 2 (67%) | | *3/44 [7%] |
