# Supplementary material for: Emergence delirium in small animals: a first step towards an objective assessment
Source: Front Vet Sci. 2025 Jun 18;12:1623761. doi: 10.3389/fvets.2025.1623761 (PMC12217935; doi:10.3389/fvets.2025.1623761)
Supplement: Supplementary file 5 [file Data_Sheet_1.docx]

Supplementary Material

# Supplementary Data

**TABLE 2_Demographic Data.**

Demographic Data of all the patients included in the study

**TABLE 4_ Symptoms.**

Table with the list of all the patients and the symptoms observed for each single patient.

# Supplementary Figures and Tables

**TABLE 1_Categories Statistics.**

Table 1 Dogs, Categories used for descriptive and inferential analysis.

Table 2 Cats, Categories used for descriptive and inferential analysis.

**TABLE 3_Demographic Data Grouped per NED/ED.**

Table 3 Dogs. Demographic and preoperative data, information regarding anesthesia, surgery and recovery phase. Data reported for dogs (from left to right) divided in the NED group and in the ED group. Round brackets refer to the percentage of animals compared to the row; square brackets refer to the percentage of animals compared to the column (total, except for "Type of surgery", for which the percentages refer only to cases undergoing a surgical procedure). * Categories added after screening the first 43 questionaries: the total number of animals needed to be adapted for these rows.

Table 4 Cats. Demographic and preoperative data, information regarding anesthesia, surgery and recovery phase. Data reported for cats (from left to right) divided in the NED group and in the ED group. Round brackets refer to the percentage of animals compared to the row; square brackets refer to the percentage of animals compared to the column (total, except for "Type of surgery", for which the percentages refer only to cases undergoing a surgical procedure). * Categories added after screening the first 43 questionaries: the total number of animals needed to be adapted for these rows
